# Epidemiological survey of anti-flea IgE in dogs in Japan by using an antigen-specific IgE quantitative measurement method

**DOI:** 10.1051/parasite/2012192173

**Published:** 2012-05-15

**Authors:** Y. Ichikawa, F. Beugnet

**Affiliations:** 1 Merial Japan Limited 2-14-2 Nagata-cho, Chiyoda-ku, Tokyo 100-0014 Japan; 2 Merial 29, avenue Tony Garnier 69007 Lyon France

**Keywords:** dog, flea, epidemiology, serology, Japan, chien, puce, épidémiologie, sérologie, Japon

## Abstract

In Japan, an epidemiological survey was performed in dogs from October to December 2008 by using a quantitative measurement method for antigen-specific IgE towards specific *Ctenocephalides felis* antigens. 214 dogs from 22 veterinary clinics were included. These clinics were located as follows, from North to South: Hokkaido, Aomori, Fukushima, Tochigi, Saitama, Chiba, Tokyo (Tama-City and Ota-ku), Kanagawa, Gifu, Niigata, Kyoto, Nara, Osaka, Hyogo, Kagawa, Ehime, Hiroshima, Yamaguchi, Fukuoka, Kumamoto and Kagoshima. 110 dogs (51.4%) were seropositive for flea-specific IgE. No differences were associated with gender or breed. This survey confirms that flea infestation in dogs is a common problem in Japan. It especially shows that the infestation also occurs in Northern Japan where fleas are considered uncommon by the vet.

## Abstract (fr)

Une étude épidémiologique a été conduite au Japon entre octobre et décembre 2008 sur des chiens par utilisation d’une méthode sérologique quantitative mesurant la réponse Ig-E anti-puces. 214 chiens ont été prélevés dans 22 cliniques vétérinaires. Ces cliniques sont situées, du nord au sud, dans les cantons de : Hokkaido, Aomori, Fukushima, Tochigi, Saitama, Chiba, Tokyo (Tama-City et Ota-ku), Kanagawa, Gifu, Niigata, Kyoto, Nara, Osaka, Hyogo, Kagawa, Ehime, Hiroshima, Yamaguchi, Fukuoka, Kumamoto et Kagoshima. 110 chiens (51,4 %) étaient séropositifs vis-à-vis des Ig-E spécifiques de l’allergène puce. Aucune différence n’est associée au sexe ou à la race. Cette étude confirme que l’infestation des chiens par les puces est un problème courant au Japon. Elle démontre également que les infestations par les puces existent dans le nord du Japon, où les puces sont parfois considérées comme absentes par les vétérinaires.

Fleas are the most common ectoparasites of both dogs and cats throughout the world (Beugnet & Franc, 2010; Farkas *et al.*, 2009). Due to their ability to reproduce in households, fleas may be present all year round, even during winter season, which explains why dogs living in cold areas may be infested. Flea bites may induce pruritus and the major pathogenic effect is flea allergic dermatitis (FAD). FAD prevention is based on the control of the flea infestation, involving regular and continued use of anti-flea drugs, which are usually topical formulations but can also be given orally (Beugnet & Franc, 2010; Farkas *et al.*, 2009; Masuda *et al.*, 2002; Medleau *et al.*, 2003). No reports on epidemiological surveys regarding flea infestations in dogs in Japan have been published. It is generally thought that the flea infestation is scarce in the Northern part of Japan due to its cold climate.

Instead of performing flea counts in order to evaluate their prevalence in dogs, which is not always easy and takes time, the authors suggested a new method to assess the flea infestation by testing the specific serological response to flea allergens. Seropositivity is a valid proof of a flea bite, even if the time (recent or old infestation) as well as the degree of infestation cannot be established.

Cat flea allergens are high-molecular proteins found in flea saliva (McDermott *et al.*, 2000). Cat flea salivary extracts also show great antigenic diversity recognized by IgE from artificially sensitized dogs or from those with FAD (Lee *et al.*, 1999). McDermott *et al.* cloned a major 18-kD allergen, called Cte f 1 in the international nomenclature (Medleau *et al.*, 2003). Its structure is very similar to that of the original molecule in terms of flea IgE-binding (Medleau *et al.*, 2003).

Intradermal testing is the standard method for identifying IgE-mediated allergic reaction *in vivo*, but it requires specific equipment and manipulation in each veterinary clinic (Laffort-Dassot *et al.*, 2004). At the opposite, specific serum IgE measurement can be performed in a well-equipped laboratory and requires only a blood sample at the veterinary clinic level. As for intradermo-reaction/prick tests, it is intended to reveal the current sensitization level to flea allergens. In this survey, serum concentration of antigenspecific IgE was quantitatively measured in ng/mL using a published technique (Okayama *et al.*, 2010). The specificity of this technique has been previously controlled by using several antigens (flea and pollens), experimental dogs that were not sensitized and experimental dogs that were sensitized. The assay enables to monitor serum antigen-specific IgE concentrations in dogs, providing information on flea exposure (Fujimura *et al.*, 2011).

## Materials and Methods

**Fig. 1.**
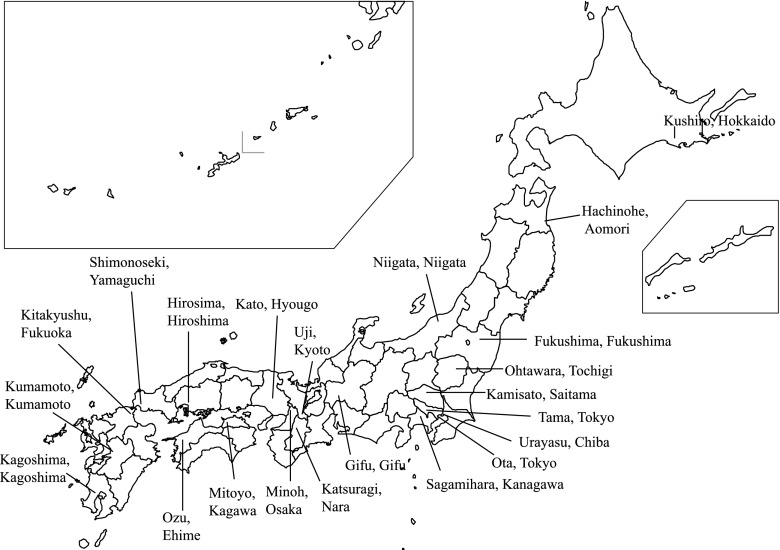
Location of the districts where the Veterinary Clinics were involved in the survey.

Serum concentration of IgE against flea antigens was measured in this epidemiological study in dogs in Japan. 22 veterinary clinics were recruited throughout Japan districts to get an estimation of flea burden on dogs (Fig. 1). All veterinary clinics were asked to randomly recruit ten dogs and collect a blood sample during the period from August to October 2008. The selected dogs had to be healthy and were not to show any clinical signs, especially dermatosis and pruritus. All dogs were client-owned and presented to the animal hospitals for routine check-ups. The age of the dogs ranged from two to seven years. A total of 214 blood samples were collected. The profiles of the dogs examined are shown in Table I.

**Table I.** Profile and IgE positive rate in the 214 examined dogs.

		Number of dogs		Anti-flea IgE positive		Anti-flea IgE titers (ng/mL)	
		n	%	n	%	Mean	SD
Total		214	100.0%	110	51.4%	43.10	85.35
Gender	Female	113	52.8%	61	54.0%	48.93	82.72
	Male	99	46.3%	48	48.5%	36.89	89.00
	Unknown	2	0.9%	1	50.0%	21.50	19.09
Breed	Miniature Dachshund	24	11.2%	15	62.5%	30.08	28.45
	Toy Poodle	17	7.9%	11	64.7%	42.65	44.83
	Chihuahua	16	7.5%	8	50.0%	45.31	48.16
	Shiba Inu	15	7.0%	5	33.3%	20.40	25.70
	Miniature Schnauzer	12	5.6%	7	58.3%	60.75	116.28
	Papillon	11	5.1%	5	45.5%	22.55	31.71
	Other pure breeds	83	38.8%	41	49.4%	48.34	90.86
	Mixed breeds	36	16.8%	18	50.0%	48.81	130.64

Serum samples from dogs were collected from October to December 2008 in the following 22 areas: Hokkaido, Aomori, Fukushima, Tochigi, Saitama, Chiba, Tokyo (Tama-City and Ota-ku), Kanagawa, Gifu, Niigata, Kyoto, Nara, Osaka, Hyogo, Kagawa, Ehime, Hiroshima, Yamaguchi, Fukuoka, Kumamoto and Kagoshima (Fig. 1).

The allergen-specific IgE quantitative measurement was performed by Animal Allergy Clinical Laboratories, Inc (Kanagawa, Japan), according to a procedure previously published (Okayama *et al.*, 2010). In short, 40 kinds of allergen-IgE including 22 environmental allergens and 18 food allergens were measured in this assay. The antigen of flea whole body was purchased from Greer Laboratories, Inc. (Lenoir, NC, USA) and used in this assay. Prior to the study, flea-specific IgE concentrations were measured in four experimental healthy Beagle dogs (two females of five-month-old and two males of four-month-old) kept in a flea-freecontrolled facility. The values obtained were respectively 0, 3, 2 and 13 ng/mL, with an average of 4.5 and a standard deviation of 5.8. The cut off was calculated as mean + 2 SD = 16 ng/mL.

Then, based on previously published (McDermott *et al.*, 2000) assessments, we estimated flea IgE positive when data showed > 16 ng/mL for flea IgE.

The total IgE half-life is considered to be short (12 hours in mice to two days in humans) (Hirano *et al.*, 1983; Tizard, 2009). However, IgE production from plasma cells seemed to continue for several months and serum antigen-specific IgE was detected during off-season of offending allergen (Masuda *et al.*, 2002; Tizard, 2009). It was therefore hypothesized that a positive result would indicate a current or past flea exposure with no possibility to a precise estimation of the flea infestation status.

Cross-reactivity of IgE against house dust mites may influence flea-IgE titers. Therefore, all the cases with coincidental sensitization to house dust mites, *i.e.* when the IgE against house dust mites (*Dermatophagoides farinae* and/or *Dermatophagoides pteronyssinus*) was higher than flea-IgE, were excluded from this study.

The statistical analysis used was the Steel-Dwass’ test with IgE values according to gender, breed (if, n > 10) and area with a significance level of p < 0.05. This survey was undertaken from October to December 2008, which only covers the end of the entire flea season. As it was not conducted throughout the whole year, a season analysis per region could not be conducted.

## Results

Table I shows the results by gender and breed. No significant differences were observed in both data sets (p > 0.05). Based on gender, 54.0% of female dogs (61/113) and 48.5% of male dogs (48/99) were positive for flea IgE. Based on breed, 62.5% of Miniature Dachshunds (n = 24), 64.7% of Toy Poodles (n = 17), 50.0% of Chihuahuas (n = 16), 33.3% of Shiba Inus (n = 15), 58.3% of Miniature Schnauzers (n = 12), 45.5% of Papillons (n = 11), 49.4% of other pure breeds (n = 83) and 50.0% of mixed breeds (n = 36) were positive for anti-flea IgE, respectively.

Positive rates of anti-flea IgE in areas examined in this study were 51.4% (110/214). Cases with anti-flea IgE were found in all 22 areas surveyed in this study. The prevalence of seropositive dogs from Northern to Southern Japan was: 30% in Hokkaido (3/10 dogs), 60% in Aomori (6/10 dogs), 10% in Fukushima (1/10 dogs), 60% in Tochigi (6/10 dogs), 80% in Saitama (8/10 dogs), 40% in Chiba (4/10 dogs), 50% in Tama-City, Tokyo (4/8 dogs), 70% in Ota-Ku, Tokyo (7/10 dogs), 50% in Kanagawa (5/10 dogs), 10% in Gifu (1/10 dogs), 10% in Niigata (1/10 dogs), 70% in Kyoto (7/10 dogs), 80% in Nara (8/10 dogs), 50% in Osaka (3/6 dogs), 70% in Hyogo (7/10 dogs), 30% in Hiroshima (3/10 dogs), 60% in Yamaguchi (6/10 dogs), 40% in Kagawa (4/10 dogs), 40% in Ehime (4/10 dogs), 30% in Fukuoka (3/10 dogs), 60% in Kumamoto (6/10 dogs) and 90% in Kagoshima (9/10 dogs). Significant differences were present between areas, regarding positive rates or mean IgE values at p < 0.05 (Table II).

**Table II.** Results of anti flea-IgE by area.

		Anti-flea IgE positive		Anti-flea IgE titers (ng/mL)	
Area	Number of dogs	n	%	Mean	SD
Kushiro, Hokkaido	10	3	30.0%	10.50	15.67
Hachinohe, Aomori	10	6	60.0%	43.90	47.67
Fukushima, Fukushima	10	1	10.0%	35.90	100.64
Ohtawara, Tochigi	10	6	60.0%	54.80	70.77
Kamisato, Saitama	10	8	80.0%	30.10	18.02
Urayasu, Chiba	10	4	40.0%	29.60	37.93
Tama, Tokyo	8	4	50.0%	84.63	124.20
Ota, Tokyo	10	7	70.0%	31.70	27.56
Sagamihara, Kanagawa	10	5	50.0%	33.30	44.40
Niigata, Niigata	10	5	50.0%	29.30	36.93
Gifu, Gifu	10	1	10.0%	4.70	6.20
Uji, Kyoto	10	7	70.0%	69.80	121.85
Minoh, Osaka	6	3	50.0%	16.50	12.41
Kato, Hyougo	10	7	70.0%	109.60	224.23
Katsuragi, Nara	10	8	80.0%	92.30	122.73
Hirosima	10	3	30.0%	13.60	15.56
Shimonoseki, Yamaguchi	10	6	60.0%	32.40	32.09
Mitoyo, Kagawa	10	4	40.0%	16.60	18.59
Ozu, Ehime	10	4	40.0%	30.50	43.11
Kitakyushu, Fukuoka	10	3	30.0%	10.40	13.37
Kumamoto, Kumamoto	10	6	60.0%	32.40	30.99
Kagoshima, Kagoshima	10	9	90.0%	133.40	158.90
Total	214	110	51,4%		

## Discussion

In all, 51.4% of dogs living in Japan were seropositive for anti-flea IgE meaning that they were either infested by fleas several weeks prior to survey dates or that they remained infested.

As expected, we did not find any significant differences in gender and breed. Significant differences were present among areas, but the interpretation of the data is difficult due to the low number of samples. It will be necessary to include more dogs in a future study, to collect samples throughout a whole year in order to evaluate the seropositivity during the four seasons, and to take into account the impact of regular or occasional anti-flea treatments.

In this time-limited study, we confirmed that dogs were often infested and bitten by fleas in Japan. We found solid proof that fleas are present in cold Northern areas, through the positivity rate of dogs that haven’t travelled around the country, despite the fact that vets and pet owners believe that fleas cannot be found in those regions. Climate change may explain the higher flea prevalence in the North, as it has also been shown for other insects. In Hokkaido, cockroaches and beetles were rarely found several decades ago but they have now increased significantly in numbers (Kida, 2007). This survey also highlights the importance of controlling fleas, given that more than 50% of dogs were infested with them. Future surveys will assess the impact of monthly flea treatments on this serological status.
